# Surgical radiofrequency ablation of atrial flutter: which operation should we choose?

**DOI:** 10.1093/jscr/rjab503

**Published:** 2021-11-11

**Authors:** Fengjie Chen, Jin Gao, Chen Song, Zhiwei Wang, Hui Xiong, Ligang Ding, Ge Gao, Hongguang Fan

**Affiliations:** Department of Cardiovascular Surgery, Fuwai Hospital Chinese Academy of Medical Sciences, Shenzhen, China; Department of Cardiovascular Surgery, Fuwai Hospital Chinese Academy of Medical Sciences, Shenzhen, China; Department of Cardiovascular Surgery, Fuwai Hospital Chinese Academy of Medical Sciences, Shenzhen, China; Department of Cardiovascular Surgery, Fuwai Hospital Chinese Academy of Medical Sciences, Shenzhen, China; Department of Cardiovascular Surgery, Fuwai Hospital Chinese Academy of Medical Sciences, Shenzhen, China; Department of Cardiovascular Surgery, State Key Laboratory of Cardiovascular Disease, Fuwai Hospital, National Center for Cardiovascular Disease, Chinese Academy of Medical Sciences and Peking Union Medical College, Beijing, China; Department of Cardiovascular Surgery, State Key Laboratory of Cardiovascular Disease, Fuwai Hospital, National Center for Cardiovascular Disease, Chinese Academy of Medical Sciences and Peking Union Medical College, Beijing, China; Department of Cardiovascular Surgery, State Key Laboratory of Cardiovascular Disease, Fuwai Hospital, National Center for Cardiovascular Disease, Chinese Academy of Medical Sciences and Peking Union Medical College, Beijing, China

## Abstract

The treatment of atrial flutter (AFL) in patients without structural heart disease (SHD) by transcatheter radiofrequency ablation of the cavotricuspid isthmus (CTI) and bilateral pulmonary veins has achieved good results. We report three cases of typical AFL treated by surgical radiofrequency ablation. One patient, without SHD, successfully underwent CTI ablation and cardioversion. The other two patients, with SHD, underwent CTI ablation, partial right atrial ablation and pulmonary vein isolation, but a normal sinus rhythm was not achieved. Therefore, standard maze IV surgery may be the best choice in patients with AFL and SHD.

## INTRODUCTION

Atrial flutter (AFL) is common in patients with structural heart disease (SHD). There is no consensus on the surgical treatment of AFL because of its complex pathogenesis, and no relevant literature has been published. However, the pathogenesis of typical AFL is relatively simple, and physicians generally use interventional catheters to ablate the cavotricuspid isthmus (CTI), with good results.

We performed surgical radiofrequency ablation in three patients with typical AFL (Table 1). Two patients, with SHD, underwent CTI plus partial right atrial ablation and pulmonary vein isolation (PVI), with cardioversion failure; the other patient, without SHD, underwent only CTI ablation, with successful sinus rhythm recovery. We report these three cases and summarize our experiences as follows.

**Table 1:** Clinical data of the patients

	Patient 1	Patient 2	Patient 3
Sex	Female	Female	Male
Age (years)	54	69	55
Preoperative diagnosis	ASD+ severe TR+ typical AFL	RHD+ moderate MS+ mild MR+ mild TR+ mild AR+ typical AFL	CHD+ typical AFL
Preoperative AFL duration (years)	3	5	1
SHD	With	With	Without
Operative methods	ASDR+ TVP+ LAAR+RFA	MVR+ TVP+ LAAR+ RFA	CABG+ RFA
Ablation route	CTI+ Partial RA+ PVI	CTI+ Partial RA+ PVI	CTI
Preoperative cardiac rhythm (Fig. 1b)	Typical AFL	Typical AFL	Typical AFL
Postoperative cardiac rhythm	Atypical AFL	Atypical AFL^*^	SR

^*^Patient 2 recovered SR after surgery and reverted to atypical AFL on the fifth day after surgery.

ASD, atrial septal defect; ASDR, atrial septal defect repair; TR, tricuspid regurgitation; RHD, rheumatic heart disease; MS, mitral stenosis; MR, mitral regurgitation; AR, aortic regurgitation; CHD, coronary heart disease; TVP, tricuspid valvuloplasty; LAAR, left atrial appendage resection; MVR, mitral valve replacements; RFA, radiofrequency ablation; CABG, coronary artery bypass grafting; RA, right atrium; SR, sinus rhythm.

## CASE REPORTS

**Figure 1:**
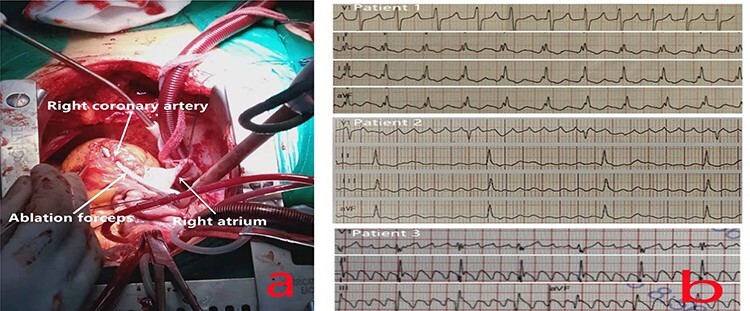
(**a**) Ablation at the midpoint of the annulus of the anterior leaflet of the tricuspid valve; to avoid injuring the right coronary artery during the operation, the right coronary artery was freed, and ablation forceps were placed under it during ablation to ensure that the ablation line extended to the tricuspid annulus and was completely transmural. (**b**) The preoperative ECG of all patients showed typical AFL.

The patients all underwent median thoracotomy, and cardiopulmonary bypass was established through the ascending aorta, superior vena cava and inferior vena cava. Bipolar radiofrequency ablation forceps (Medtronic, Inc., Michigan, USA) were used for radiofrequency ablation. The ablation route in patients 1 and 2 included bilateral PVI, the midpoint of the border crest of the incision in the right atrium to the midpoint of the annulus of the anterior leaflet of the tricuspid valve (Fig. 1a), the border crest side of the right atrium to the inferior vena cava orifice, and the tricuspid valve ring side of the right atrium to the front end of the right atrial appendage. Only CTI ablation was performed in patient 3.

## DISCUSSION

Typical AFL is a type of macroreentrant atrial tachycardia. The most common type of typical AFL is CTI-dependent AFL, in which electric stimulation is conducted around the tricuspid annulus in a large reentrant loop with the CTI as the lower boundary of the reentrant loop [1]. Atypical AFL is a narrative term for atrial tachycardia; its pathogenesis is complex and not yet fully clear. In general, atypical AFL is CTI-independent macroreentrant atrial tachycardia [1].

In typical AFL, the reentrant loop is mostly CTI dependent. Theoretically, most reentrant loops can be successfully interrupted by the ablation of a right atrial maze.

Patient 3, in whom only CTI ablation was performed, showed successful sinus rhythm conversion. Although typical AFL was interrupted successfully in patients 1 and 2, atypical AFL eventually developed. Additionally, even though PVI was also performed, we still failed to achieve a permanent sinus rhythm in these two cases. Related studies have also shown that after the successful elimination of a simple typical AFL by catheter radiofrequency ablation, 82% of patients will experience at least one new episode of atrial fibrillation (AF) during the follow-up period [2]. Mohanty *et al*. [3] reported that for patients with simple AFL without AF, adding PVI to CTI can increase the rate of sinus rhythm maintenance (71.3 vs. 60.2%). The short-term and long-term results are mainly related to the diameter of the left atrium; the larger the diameter of the left atrium, the lower the rate of success [2].

The two patients with SHD in this report had a long history of AFL, and CTI ablation plus partial right atrial ablation and PVI failed to restore the sinus rhythm permanently. The pathological mechanism of AFL caused by severe SHD may be more complicated than previously thought, which may be related to abnormal electrical conduction in different parts of the heart or to focal excitability [4]. The presence of SHD can also affect the distinction between focal tachycardia and macroreentrant atrial tachycardia [5], thereby affecting the choice of the correct surgical approach. AFL and AF have the same risk factors, such as valvular heart disease, cardiac insufficiency and hypertension. AFL and AF also have an inseparable relationship in terms of pathogenesis. Atrial electrical remodeling and anatomical remodeling are common triggering factors and maintenance matrices. Under certain conditions, AFL and AF can transform into each other [6].

In addition, although typical AFL has very stable electrophysiological and anatomical characteristics [7], the electrocardiogram (ECG) morphology is also regular. However, even if the ECG shows typical AFL, it is not necessarily CTI dependent [8]. There may be a reentry mechanism involving the left atrium, coronary sinus and other areas. According to our experience, in patients with AFL and SHD, even CTI plus partial right atrial ablation and PVI cannot cure AFL. Perhaps standard maze IV surgery is the only way to interrupt reentry beyond the right atrium and increase the rate of success.

In summary, in patients with severe SHD, even those with a stable typical AFL confirmed by ECG, to increase the success rate of the operation, a complete standard maze IV operation may be the best treatment strategy.

## CONFLICT OF INTEREST STATEMENT

None declared. No medical ethics were involved in this study. All patients provided written informed consent.

## FUNDING

Shenzhen Key Medical Discipline Construction Fund (No. SZXK019).
